# Optimizing Cell-Free Protein Synthesis for Increased Yield and Activity of Colicins

**DOI:** 10.3390/mps2020028

**Published:** 2019-04-11

**Authors:** Xing Jin, Weston Kightlinger, Seok Hoon Hong

**Affiliations:** 1 Department of Chemical and Biological Engineering, Illinois Institute of Technology, Chicago, IL 60616, USA; xjin14@hawk.iit.edu; 2 Department of Chemical and Biological Engineering, Northwestern University, Evanston, IL 60208, USA; westonkightlinger@u.northwestern.edu

**Keywords:** colicins, cell-free protein synthesis, antimicrobials, chaperones

## Abstract

Colicins are antimicrobial proteins produced by *Escherichia coli* that hold great promise as viable complements or alternatives to antibiotics. Cell-free protein synthesis (CFPS) is a useful production platform for toxic proteins because it eliminates the need to maintain cell viability, a common problem in cell-based production. Previously, we demonstrated that colicins produced by CFPS based on crude *Escherichia coli* lysates are effective in eradicating antibiotic-tolerant bacteria known as persisters. However, we also found that some colicins have poor solubility or low cell-killing activity. In this study, we improved the solubility of colicin M from 16% to nearly 100% by producing it in chaperone-enriched *E. coli* extracts, resulting in enhanced cell-killing activity. We also improved the cytotoxicity of colicin E3 by adding or co-expressing the E3 immunity protein during the CFPS reaction, suggesting that the E3 immunity protein enhances colicin E3 activity in addition to protecting the host strain. Finally, we confirmed our previous finding that active colicins can be rapidly synthesized by observing colicin E1 production over time in CFPS. Within three hours of CFPS incubation, colicin E1 reached its maximum production yield and maintained high cytotoxicity during longer incubations up to 20 h. Taken together, our findings indicate that colicin production can be easily optimized for improved solubility and activity using the CFPS platform.

## 1. Introduction

Multidrug-resistant bacteria can be difficult to treat and are a serious threat to society [1]. There is an immediate need for the development of new antimicrobial drugs to counteract the increase in drug-resistant pathogens and the weakness of current antibiotic discovery pipelines [2]. A subgroup of antimicrobial peptides/proteins known as bacteriocins are considered to be viable alternatives to antibiotics because they exhibit high cell-killing activity against clinically important pathogens (both in vivo and in vitro), low oral toxicity to the host, as well as both broad- and narrow-spectrum qualities. Furthermore, bacteriocins can be produced by probiotic bacteria and easily bioengineered via protein engineering [3].

Antimicrobial peptides are short (5~100 amino acids) and can be synthesized either chemically or biologically [4]; however, the complete chemical synthesis of high-molecular weight proteins is challenging and generally require biological production by expression of corresponding genes in a host strain or in vitro system [5]. Compared to cell-based protein production, cell-free protein synthesis (CFPS) provides several advantages for producing toxic proteins, as demonstrated in the cases of onconase (RNase) [6], pierisin-1b [7], cecropin P1 [8], and colicins [9], which are deleterious to the host cells when overproduced. Most importantly, CFPS platforms do not need to maintain host cell viability during protein production because the transcriptional and translational machineries have already been extracted from lysed cells [10]. In addition, CFPS platforms provide an open reaction environment that can be easily controlled and optimized [11,12]. Our group recently demonstrated that colicins, bacteriocins produced by *Escherichia coli*, can be produced using an *E. coli*-based CFPS system [9]. Colicins kill non-host *E. coli* cells [13] by inhibiting cell wall synthesis (e.g., colicin M) [14], forming pores in inner membrane (e.g., colicin E1 [15] and Ia [16]), and degrading DNA (e.g., colicin E2) [17] or RNA (e.g., colicin E3) [18]. We reported that colicins E1 and E2 are very effective in killing antibiotic-tolerant persister cells [9]. However, some colicins such as colicin M exhibited low solubility and poor cell-killing activity when produced in CFPS. Therefore, further improvement of colicin production and bioactivity is required for optimal cell-free colicin production.

When the proteins are not folded properly, they become insoluble and form inclusion bodies in the cell [19]. Because proper three-dimensional protein folding is critical to achieve full protein function, these insoluble proteins are generally inactive [20]. Several strategies are available to improve solubility and thereby enhance protein folding including reducing protein synthesis rate, changing the growth medium, co-expressing molecular chaperones or foldases, and adding fusion partners to the target protein [21]. Similar approaches have been applied to produce ‘difficult-to-express’ proteins in vitro by harnessing the open and flexible nature of the CFPS platform [10]. Molecular chaperones prevent protein aggregation and promote protein folding via ingenious mechanisms [22]. The exogenous addition of molecular chaperone proteins has successfully facilitated the solubility of hundreds of proteins in cell-free translation system [23,24]. Common examples of molecular chaperones that can be used to prevent protein aggregation and misfolding include the GroES/EL and DnaK/DnaJ/GrpE chaperone systems [25]. For instance, cell extracts enriched with GroES/EL chaperones have been used to increase the yield of functional antibody fragments [26], and the solubility of the human erythropoietin was dramatically enhanced by cell extracts enriched with DnaK/DnaJ/GrpE chaperones [27]. Based on these previous works, we reasoned that CFPS could be optimized to improve colicin production yields and activity.

In this study, we investigated whether the production of colicins exhibiting low solubility, low yield, or low activity can be improved by optimizing CFPS lysates and reaction conditions. Here we report that the solubility of colicin M can be improved from 16% to nearly 100% by using CFPS lysates enriched with chaperones and that the cell-killing activity of colicin E3 can be increased by five orders of magnitude by co-expressing its immunity protein in the CFPS reaction. In addition, we measured the kinetics of colicin E1 synthesis and observed rapid formation of active colicin in CFPS reactions. This work provides new strategies to produce high titers of active colicins in CFPS and finds that CFPS is an excellent biological platform for the production and optimization of toxic proteins.

## 2. Materials and Methods

### 2.1. Bacterial Strains and Plasmids

The bacterial strains and plasmids used in this study are found in Table 1. We used BL21 Star (DE3) to prepare crude extracts because the strain carries a genomic copy of T7 RNA polymerase and a mutation in the RNase E gene (*rne131*) which facilitate the transcription and prevent the degradation of messenger RNAs, respectively [28], during the CFPS reaction [29]. We used the *E. coli* K361 strain for testing colicin cell-killing activity [30]. Plasmids containing molecular chaperone genes were purchased (Takara Bio, Shiga, Japan). These plasmids carry an origin of replication derived from pACYC and a gene providing chloramphenicol resistance. The chaperone genes are controlled by the *araB* or *Pzt-1* promoters which are induced by L-arabinose or tetracycline, respectively (Table 1). Detailed plasmid information can be found in the product manual provided from Takara Bio. Streptomycin (100 μg/mL), kanamycin (50 μg/mL), ampicillin (100 μg/mL) and chloramphenicol (35 μg/mL) were added in the cell culture to maintain plasmids as necessary.

### 2.2. Crude Extract Preparation

*E. coli* crude extracts for CFPS were prepared from the BL21 Star (DE3) strain using a sonication method [31] as described previously [9]. Chaperone-enriched cell extracts were prepared with similar methods after induction of chaperone protein production during *E. coli* cultivation. An overnight culture of BL21 Star (DE3) containing chaperone plasmids was diluted 1,000 times in 1.0 L of 2xYTPG medium (16 g/L tryptone, 10 g/L yeast extract, 5 g/L NaCl, 7 g/L K_2_HPO_4_, 3 g/L KH_2_PO_4_, and 18 g/L glucose; pH 7.2 adjusted with KOH) with appropriate antibiotics in a 2.5 L Tunair flask. Cells were grown to a turbidity at 600 nm (OD_600nm_) of 0.5 at 37 °C at 220 rpm. The GroES/EL chaperone system from pGro7 was induced by adding 0.5 mg/mL L-arabinose. Similarly, DnaK/DnaJ/GrpE system from pKJE7 was induced by adding 0.5 mg/mL L-arabinose. For cell cultures containing both chaperone systems from pG-KJE8, GroES/EL was induced by adding 1 ng/mL tetracycline and DnaK/DnaJ/GrpE was induced by adding 0.5 mg/mL L-arabinose. After inducing chaperones, the cells were further grown to an OD_600nm_ of 3.0. Then, cells were harvested by centrifuging at 5,000× *g* at 4 °C for 15 min, washed twice to remove all the medium with cold S30 buffer (10 mM tris-acetate pH 8.2, 14 mM magnesium acetate, 60 mM potassium acetate, 1 mM dithiothreitol), and stored at −80 °C. Thawed cells were mixed with S30 buffer (1 mL buffer per 1 g of cells) and lysed on ice using a Q125 sonicator (Qsonica, Newtown, CT, USA) using 50% amplitude and three cycles of 45 s pulses at 60 s intervals. Insoluble components including cell debris were removed by two centrifugation steps at 14,000× *g* at 4 °C for 10 min. The crude extracts were filtered by a 0.2 μm sterile syringe filter (Corning, Corning, NY, USA) to remove unlysed cells completely. The total protein concentration of the extracts was approximately 50 mg/mL, as assessed by Quick-Start Bradford protein assay kit (Bio-Rad, Hercules, CA, USA). The crude extracts were stored at −80 °C until needed.

### 2.3. Preparing Linear DNA Template for Cell-Free Colicin Production

Colicin genes were amplified by polymerase chain reaction (PCR) using primers found in Table S1. The *cma* gene encoding colicin M was amplified from the pJL1-*cma* plasmid using GAcol-F and GAcol-R, *ceaC* encoding colicin E3 from the pKSJ167 plasmid using ColE3-F and ColE3-R, and *cea* encoding colicin E1 from the pKSJ331 plasmid using ColE1-F and ColE1-R. PCR was performed using Phusion High-Fidelity DNA polymerase (New England Biolabs, Ipswich, MA, USA) at 98 °C for 30 s, with 30 cycles of denaturing at 98 °C for 10 s, annealing at 55 or 60 °C for 30 s, and extending at 72 °C for 2 min 30 s, and a final extension at 72 °C for 10 min. Three rounds of PCR were performed to insert T7 promoter and T7 terminator sequences for genes of colicin M, E1, and E3. Phosphorothioated T7Mega-F and T7Mega-R primers were used to protect T7 promoter and terminator sequences from nuclease degradation. The first PCR amplified the colicin genes, the second PCR added the T7 promoter sequence, and the third PCR added the T7 terminator sequence. DNA sequences of all colicin genes are found in Table S2. The PCR products were purified using E.Z.N.A. Cycle Pure kit (Omega Bio-Tek, Norcross, GA, USA) before addition to CFPS reactions.

### 2.4. Preparing E3 Immunity Protein

The E3 immunity gene *imm* was cloned into the pJL1 plasmid. First, the gene was amplified from the pKSJ167 plasmid using the primers E3Imm-F and E3Imm-R which installed a C-terminal Strep-tag (WSHPQFEK). The PCR fragment was double digested by NdeI and SalI restriction enzymes and ligated into pJL1-backbone at the same restriction sites. BL21 Star (DE3) cells were transformed with the ligated plasmid. An overnight culture of *E. coli* BL21 Star (DE3) harboring the pJL1-E3 *imm* plasmid was diluted 100 times in 250 mL of LB containing 50 μg/mL of kanamycin in a 1 L flask and incubated at 37 °C at 220 rpm until OD_600nm_ ~ 0.5. Then, E3 immunity protein production was induced by adding 1 mM of isopropyl β-d-1-thiogalactopyranoside at 37 °C at 220 rpm for an additional 5 h. E3 immunity protein was purified by a Strep-Tactin column (IBA, Göttingen, Germany), and the concentration of the E3 immunity protein was measured as 520 ± 30 μg/mL.

### 2.5. CFPS Reaction

CFPS reactions were performed to synthesize colicins according to established protocols [9]. Chaperone-enriched or standard BL21 Star (DE3) extracts were used, and RNase Inhibitor (New England Biolabs) or purified E3 immunity protein were added as necessary. The CFPS samples (15 μL in a 1.5 mL microcentrifuge tube) were incubated for up to 20 h at 30 °C or room temperature (~25 °C).

### 2.6. Quantifying Colicins Using Radioactive ^14^C-Leu Assay

Total and soluble protein yields were measured by determining radioactive ^14^C-Leu incorporation [35]. Briefly, 10 µM ^14^C-leucine (Perkin-Elmer, Waltham, MA, USA) was added into triplicate CFPS reactions. After incubating at 30 °C or room temperature for specified periods of time, soluble fractions were separated by centrifuging at 12,000× *g* for 15 min at 4 °C. CFPS reactions in the time-course study of colicin E1 synthesis were quenched at indicated times using 833 µg/mL kanamycin and flash freezing at −80 °C. Proteins were precipitated, washed with 5% trichloroacetic acid three times and then 100% ethanol, and quantified using a liquid scintillation counting. To eliminate background radioactivity and protein synthesis, scintillation counts from no plasmid control were subtracted from the colicin samples. For colicin M, total or soluble fractions of each reaction containing ^14^C-leucine were visualized by running an SDS-PAGE gel, exposing the gel Storage Phosphor Screen, and acquiring an autoradiogram using a Typhoon FLA700 imager as described previously [9].

### 2.7. Cell Viability Test

Cell viability assays were performed as described previously [9]. An overnight culture of the K361 indicator cells was regrown in fresh LB medium at 220 rpm at 37 °C until an OD_600nm_ of approximately 0.7–0.9. Cells were harvested, adjusted to an OD_600nm_ of 0.1 (equivalent to 5.0 × 10^7^ CFU/mL) or 1.0 (equivalent to 5.0 × 10^8^ CFU/mL) with LB medium, and then incubated with CFPS reaction products for 1 h with shaking at 37 °C. Cell viability was quantified by counting colony forming units. To generate the activity curve of colicin E3, we treated cells at high initial population (5.0 × 10^8^ CFU/mL) with varying concentrations of colicin E3 for 1 h. Effective multiplicity (m), a measure of colicin cytotoxicity, was calculated by the equation: m = −ln(S/S_0_), where S indicates the surviving cell population with colicin treatment, and S_0_ is the untreated control cell population [36].

### 2.8. Statistical Analysis

Two-tailed *t*-tests between colicin samples and no colicin controls were performed [9]. Statistical significance is indicated in figures with * (*p* < 0.01), ** (*p* < 0.001), and *** (*p* < 0.0001).

## 3. Results and Discussion

### 3.1. Enrichment of Cell Extracts with Chaperones Does Not Significantly Affect CFPS Productivity

While our previous study showed that colicins E1, E2 and Ia were nearly completely soluble when produced in CFPS, we found that the majority of colicin M produced was insoluble and that only soluble colicin M (~5%) was active in killing K361 indicator cells [9]. Based on previous reports that cell extracts enriched with chaperones and disulfide bond isomerases can enhance the production of functional antibodies in CFPS [26], we sought to apply a similar strategy to improve the solubility of colicin M. We overexpressed three sets of molecular chaperones (GroES/EL (Gro), DnaK/DnaJ/GrpE (KJE), and both GroES/EL and DnaK/DnaJ/GrpE (Gro-KJE)) in BL21 Star (DE3) *E. coli* cultures and then harvested and prepared extracts from these chassis strains using a sonication method [31] followed by the removal of unlysed cells by syringe filtration [9]. First, we examined if the overexpression of chaperones in CFPS extract chassis strains affected their overall in vitro protein production capacity by producing superfolder green fluorescent protein (sfGFP) (Figure S1). sfGFP is known as a highly soluble protein with rapid folding kinetics [37] and has been widely used as an indicator to examine the CFPS capacity of cell extracts [31]. The sfGFP production levels using the chaperone-enriched cell extracts were similar (within ± 20% difference) to the sfGFP level using the standard cell extract (Star) prepared from BL21 Star (DE3) without chaperone overexpression. As expected, we obtained almost 100% soluble sfGFP for all extracts tested. These results show that chaperones in the extracts do not strongly affect transcription and translation during CFPS reactions.

### 3.2. Solubility of Colicin M is Increased in the Presence of Chaperones in CFPS

**Figure 1:**
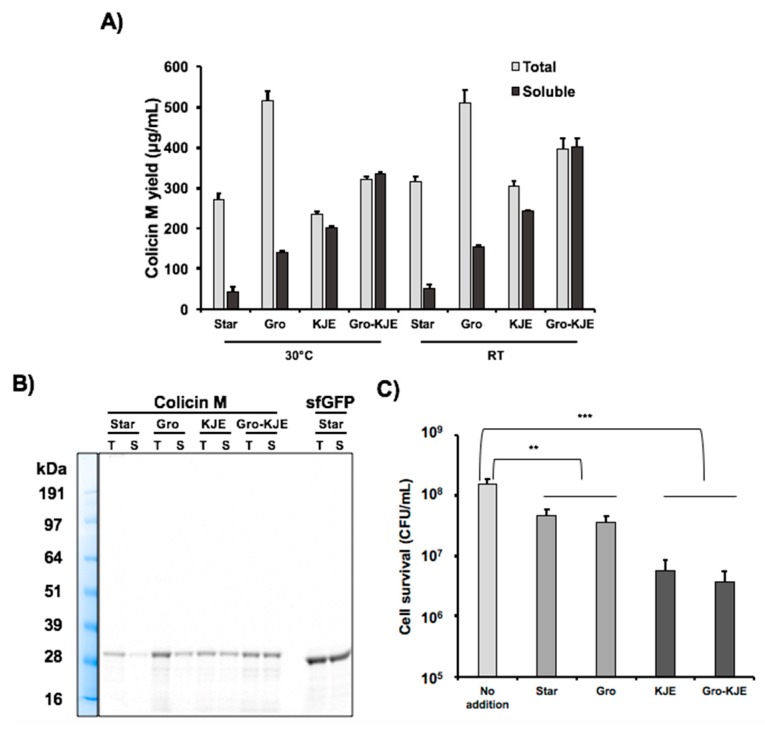
Improvement of colicin M production in cell-free protein synthesis (CFPS). (**A**) Total and soluble protein yield for cell-free produced colicin M with molecular chaperone-enriched extracts quantified by ^14^C-Leu scintillation counting at 30 °C and room temperature (RT). Cell extract without chaperone production was prepared from BL21 Star (DE3) strain (Star). Chaperone-enriched extracts were prepared from BL21 Star (DE3) cultures overexpressing GroES/EL (Gro), DnaK/DnaJ/GrpE (KJE), and both GroES/EL and DnaK/DnaJ/GrpE (Gro-KJE) in BL21 Star (DE3). Error bars indicate standard deviations from three independent CFPS reactions. (**B**) Radioactive ^14^C-Leu autoradiogram gel of total (T) and soluble (S) protein yield for colicin M and sfGFP produced during CFPS reactions with different cell extracts. (**C**) Viability of K361 indicator cells. K361 cells (initial cell density 5 × 10^7^ CFU/mL) were treated with 750 ng/mL total concentration of colicin M produced by CFPS using different chaperone-enriched extracts and then incubated at 37 °C and 220 rpm for 1 h. Error bars indicate standard deviation from two independent cultures with three plating replicates each. ** and *** represent significant difference compared to no addition sample under the same media conditions with *p*-values of < 0.001 and <0.0001, respectively.

Next, we produced colicin M by CFPS using the three chaperone-enriched extracts characterized above to determine if the chaperones could enhance solubility of colicin M. We incubated the cell-free reactions for 20 h at 30 °C and then quantified total and soluble colicin M production via radioactive ^14^C-Leu incorporation. Total colicin M yield was approximately 300 ng/μL in all extracts except the GroES/EL-containing extract which yielded approximately 500 ng/μL (Figure 1A). The total colicin M yield in CFPS was comparable with other colicins (E1, E2, and Ia) previously reported [9]. Without chaperone-enriched cell extracts, less than 20% of colicin M was soluble. However, the Gro chaperone system increased the solubility to close to 30%, and the KJE chaperone system improved the solubility to more than 80%. When both chaperone systems were present in the cell extract (Gro-KJE), colicin M was completely soluble (Table 2, Figure 1A). The improvement of colicin M solubility by chaperones present in the CFPS reaction was confirmed by an SDS-PAGE gel autoradiogram (Figure 1B). Our results reveal that GroES/EL and DnaK/DnaJ/GrpE chaperones overexpressed in CFPS chassis strains can assist in protein folding in vitro, thereby enhancing the colicin M solubility and the titers of soluble colicin M which can be produced during cell-free reactions. Notably, the presence of both chaperone systems results in a synergistic effect leading to the production of colicin M that is 100% soluble.

Although we did not purify colicin M from the cell-free reaction that contains chaperones and other CFPS components, we expect that colicin M is still soluble and active when the chaperones are removed via purification. Previous work suggests that the molecular chaperones used in our cell-free reactions form protein-chaperone complexes to assists in folding, and that after folding, the protein is released from the complex to become a folded or native protein [38,39]. Another report demonstrated that a soluble single chain variable fragment (scFv) antibody can be successfully purified after co-expression with a molecular chaperone [40].

Decreasing incubation temperatures in cell cultures producing heterologous proteins is known to result in decreased protein aggregation [41]. This is likely due to slower protein production rates at lower temperatures which give newly translated proteins time to fold properly [21]. To examine whether or not lowering the temperature during protein synthesis could further influence colicin M production, we incubated cell-free reaction with and without molecular chaperones at room temperature (~25 °C) for 20 h. The incubation temperature did not affect colicin M solubility (Table 2), but the soluble colicin M production yields were improved 17 ± 5% at room temperature compared to the colicin M yield at 30 °C (Figure 1A). Similar low temperature effect in enhancing protein yield in CFPS was also reported in sfGFP synthesis [33] and ribosomal RNA synthesis and ribosome assembly [42]. Since the amount of soluble colicin M yield was increased at the lower incubation temperature, we produced colicin M via CFPS at room temperature for later study. Taken together, our data shows that cell extracts enriched with molecular chaperones enhance colicin M solubility and lowering the incubation temperature from 30 °C to room temperature increases colicin M yields.

### 3.3. Increased Colicin M Solubility Enhances Cell-Killing Activity

As the colicin M solubility was increased with the chaperone-enriched extracts, we investigated the cell-killng activity of colicin M produced under these conditions. To prepare indicator cells for this experiment, a culture of *E. coli* K361 strain was incubated to reach exponential growth phase with a turbidity (OD_600nm_) between approximately 0.7–0.9, centrifuged, and resuspended in fresh LB to adjust cell populations to approximately 5.0 × 10^7^ CFU/mL. The indicator cells were exposed to a 750 ng/mL total concentration of cell-free produced colicin M for 1 h at 37 °C with shaking at 220 rpm (Figure 1C). We observed that colicin M produced by chaperone-enriched cell extracts (KJE and Gro-KJE extracts) lowered surviving cells up to 12-fold compared to the colicin M produced by the Star extract. The levels of cell survival observed in cultures treated with colicin M produced by KJE extract was similar to the cultures treated with colicin M produced by Gro-KJE extract (Figure 1C). Because the KJE extract already increased the colicin M solubility to over 80%, the solubility improvement to 100% achieved by using Gro-KJE extract (Table 2) might not result in significant differences in the apparent cell-killing activity of colicin M. We previously reported that only soluble colicin M exhibited cell-killng activity [9], likely due to the improper folding and therefore a lack of activity of the insoluble proteins [19]. These results suggest that the increased solubility of colicin M achieved by production in lysates enriched with molecular chaperones (KJE and Gro-KJE extracts) enhances the overall cell-killing activity of colicin M.

### 3.4. Co-Expression of Colicin E3 and Its Immunity Protein Enhances E3 Activity

**Figure 2:**
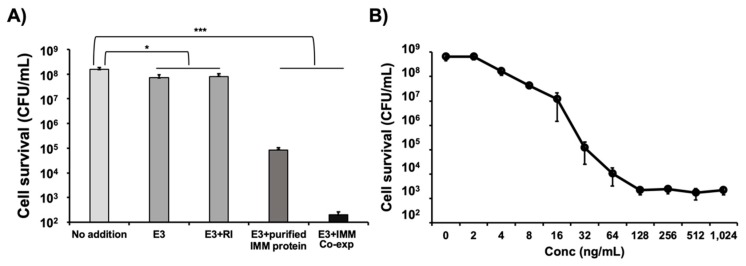
Improvement of colicin E3 activity in CFPS. (**A**) Viability of K361 indicator cells (initial cell density 5 × 10^7^ CFU/mL) upon treatment with 250 ng/mL of cell-free produced colicin E3 with 2.7 U/μL RNase inhibitor, 16.7 ng/μL immunity protein, and co-expression (Co-exp) of immunity protein at 37 °C 220 rpm for 1 h. (**B**) Effect of increasing concentrations of cell-free produced colicin E3 with co-expression of immunity protein on K361 cells (initial cell density 5 × 10^8^ CFU/mL) at 37 °C 220 rpm for 1 h incubation. Error bars indicate standard deviation from two independent cultures with three plating replicates each. * and *** represent significant differences compared to no addition sample under the same media conditions with *p*-values of < 0.01 and <0.0001, respectively.

We previously reported that the production of colicin E2 (which has DNase activity) in CFPS does not require the addition or co-expression of its immunity protein. In this study, we were interested in producing another colicin (E3) that is known to have a specific RNase activity [18] which degrades the 16S ribosomal RNA [43]. We originally hypothesized that because of this RNase activity, colicin E3 would be difficult to produce in cell-free as it might digest the ribosomes which are required for its synthesis and that blocking this RNase activity during its CFPS production would increase overall yields. We did, in fact, observe low production yield of colicin E3 (~60 ng/μL) (Figure S2) which was 5-fold lower than that of colicin E1 and E2 (~300 ng/μL) in CFPS [9]. However, neither the addition of RNase inhibitor [44] nor the addition of exogenously produced E3 immunity protein (both of which would presumably block the RNase activity of colicin E3) were effective in increasing our overall yields (Figure S2). We also attempted to co-express the E3 immunity gene together with the colicin E3 gene in an operon during CFPS, but this co-expression also had no effect on the protein production yield (Figure S2). Despite the fact that the addition of RNase inhibitor and E3 immunity protein did not improve production yields, we decided to test the cytotoxicity of colicin E3 produced in CFPS with exogenous addition of RNase inhibitor or its immunity protein or co-expression of E3 and immunity protein (Figure 2A). The cell-killng activity of colicin E3 synthesized without the immunity protein or supplemented with RNase inhibitor present during the CFPS reaction possessed very little cell-killing activity. However, we were surprised to find that the exogenous addition of the E3 immunity protein into the CFPS reaction increased the E3 activity 1000-fold and that the co-expression of colicin E3 and immunity protein during the cell-free reaction increased the E3 activity 10^5^-fold with a multiplicity of 13.5, killing nearly all indicator cells (Figure 2A).

Our results suggest that the immunity protein is necessary for colicin E3 to maintain its activity. While initially unexpected, this finding makes sense in the context of a structural study of colicin E3 [45] which found that the colicin E3 protein alone contains a disordered cytotoxicity domain that is restored to its native structure when complexed with the E3 immunity protein. We therefore conclude that colicin E3 produced alone in CFPS has very low activity due to a disordered cytotoxicity domain and that this cytotoxicity domain folds into its native structure and becomes active when the colicin E3 immunity protein is added to the reaction. Furthermore, we observed that co-expression of the immunity protein provided much more highly active colicin E3 compared to the addition of purified immunity protein. This difference suggests that there may be a close interaction between colicin E3 and the immunity protein that is required during their folding to obtain full activity from the complex. Using the co-expression approach, we produced fully active colicin E3 and assessed the cell-killing activity by varying concentrations of E3. Maximum cell-killng was achieved at concentrations above 128 ng/mL of E3 and the immunity complex with a multiplicity around 13 (Figure 2B) which is comparable to the activity of cell-free synthesized colicins E1 and E2 [9] and consistent with the previous report that the multiplicity of colicin E3 produced in vivo was 13.9 at a concentration of 3.2 nM (equivalent to 188 ng/mL) with initial cell population around 5.0 × 10^7^ CFU/mL for 1 h [36]. Taken together, our data suggests that the cell-killing activity of cell-free synthesized colicin E3 can be drastically improved by addition or co-expression of the colicin E3 immunity protein to the CFPS reaction to levels that are comparable to those of colicin E3 produced in cells.

### 3.5. Colicin E1 Is Rapidly Produced and Remains Stable in CFPS

Because CFPS reactions can be lyophilized, stored without cold-chain, and rehydrated to provide simplified and rapid access to high yields of proteins [46], on-demand or distributed manufacturing is a promising application area for CFPS [47]. Production of native or engineered colicins in vitro can be applied to quickly kill a wide variety of pathogens common in resource-limited settings. However, speed of production and protein stability will be critical parameters for these applications and there is currently little information regarding the kinetics of colicin synthesis in CFPS.

**Figure 3:**
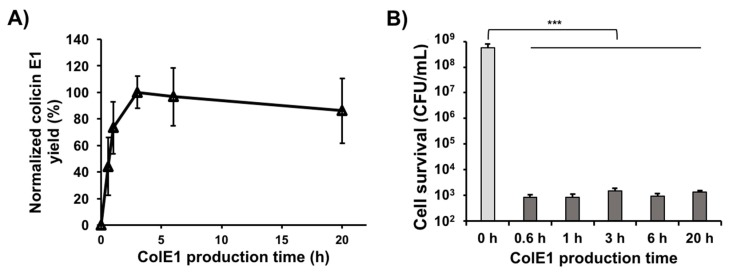
Cell-free production kinetics of colicin E1. (**A**) Yields of soluble colicin E1 produced in CFPS over time. Yields were determined by incorporation of radioactive ^14^C-leucine and normalized to the maximum yield observed after 3 h incubation. (**B**) Viability of K361 cells (initial cell density 5 × 10^7^ CFU/mL) upon treatment with 250 ng/mL of soluble cell-free produced colicin E1 at 37 °C 220 rpm for 1 h. Error bars indicate standard deviation from two independent cultures with three plating replicates each. *** represents significant difference compared to 0 h sample under the same media conditions with *p*-value < 0.0001.

In our previous study, colicin Ia produced in CFPS reached 80% of its maximum concentration after just 1 h, reached its maximum concentration at 3 h, and then maintained approximately the same concentration over the remainder of the 20 h incubation time [9]. To investigate earlier time-points and see if such rapid colicin production can be applied to other colicins, we produced colicin E1 in CFPS with 200 ng of linear PCR template and monitored soluble colicin E1 yield over time using ^14^C-Leu scintillation counting (Figure 3A). Kanamycin was added to the cell-free reaction to a final concentration of 833 μg/mL and the reactions were flash frozen on liquid nitrogen to immediately stop translation after the designated time. Consistent with colicin Ia production [9], soluble colicin E1 was produced rapidly at early time points (until 3 h when it reached its highest concentration) and then maintained approximately the same concentration over the time course (until 20 h). We also performed cell viability assays by treating an initial K361 indicator cell population of 5.0 × 10^8^ CFU/mL with 250 ng/mL of colicin E1 produced in CFPS reactions incubated for various amounts of time. The cell-free produced colicin E1 exhibited very high killing activity and the activities were similar across various CFPS incubation times (Figure 3B), indicating that active colicin E1 is produced rapidly in the CFPS reaction and that up to 20 h, increased incubation time in the CFPS reaction has a negligible effect on colicin E1 cytotoxicity. Taken together with the previous study [9], these results indicate that active colicins can be produced in just 30 mins, reach their maximum yields at approximately 3 h, and that longer incubation times in the CFPS reactions do not adversely affect the activity of colicins.

The rapid and robust production of colicin E1 reported here supports further research into the possible utility of CFPS production of colicins for on-demand protein manufacturing. Because colicins kill non-host *E. coli* cells by recognizing receptors on the cell surface [13], they may be effective in combating *E. coli* strains which cause infectious diseases including urinary tract infection, sepsis/meningitis, and enteric/diarrheal disease [48]. Although effective dosages of colicin E1 in humans have not been determined, a rough estimation of required dosages also suggests that the small-scale production of single dosages of colicin E1 by CFPS is feasible. Fluoroquinolones or third-generation cephalosporins are commonly used to treat many bacterial infections, including those listed above [49]. The FDA has reported that the antibiotic ciprofloxacin (a type of fluoroquinolone) with a MIC_90_ (minimum inhibitory concentration that kills 90% of cell population) of 1.0 μg/mL requires an oral dosing of 250~750 mg per 12 h [50]. Using ciprofloxacin as a guide, the amount of colicin E1, which has a calculated MIC_90_ of 0.016 μg/mL [9], required for a single oral dose with equivalent efficacy can be estimated to be approximately 0.4–1.2 mg. We have found that colicin E1 can be produced at 0.3 mg/mL using CFPS. Therefore, the amount of CFPS reaction volume required to make 1 mg of colicin E1 (approximately one oral dose) would be 3.3 mL, a reasonable volume for synthesis and dosing. Previous studies have shown that total protein yields in *E. coli* CFPS reactions can reach 2.3 mg/mL for sfGFP [51], indicating that additional optimization of CFPS conditions could further decrease the volume of CFPS required for on-demand protein manufacturing of colicins.

## 4. Conclusions

In summary, we utilized the open nature and engineering flexibility of CFPS to improve colicin M solubility with chaperone-enriched cell extracts, enhance the activity of colicin E3 via co-expression of the immunity protein, and assess the production kinetics and stability of colicin E1 in CFPS reactions. We anticipate that the approaches presented in this study can be applied to optimize the production of other colicins or colicin-like bacteriocins in vitro, providing a useful alternative to the in vivo production of toxic proteins.

## Figures and Tables

**Table 1:** Strains and plasmids used in this study. Str^R^, Km^R^, Am^R^, and Cm^R^ are streptomycin, kanamycin, ampicillin, and chloramphenicol resistance, respectively.

Strains and Plasmids	Genotype/Relevant Characteristics	Source
**Strains**		
*E. coli* K361	Wild type W3110 strain with Str^R^	[30]
*E. coli* BL21 Star (DE3)	F^−^ *ompT*, *hsdS_B_* (r_B_^−^m_B_^−^), *gal*, *dcm*, *rne131* (DE3)	Invitrogen
*E. coli* TG1	Strain containing colicin plasmid	[32]
**Plasmids**		
pJL1-sfGFP	Km^R^, *P_T7_*::s*fGFP*, C-terminal Strep-tag	[33]
pJL1-*cma*	Km^R^, *P_T7_*::*cma* encoding colicin M	[9]
pJL1-E3 *imm*	Km^R^, *P_T7_*::*E3imm* encoding E3 immunity	This study
pKSJ331	Am^R^, ColE1 operon	[32]
pKSJ167	Am^R^, ColE3 operon	[34]
pGro7	Cm^R^, *P_araB_*::*groES-groEL* encoding GroES/EL	Takara Bio
pKJE7	Cm^R^, *P_araB_*::*dnaK-dnaJ-grpE* encoding DnaK/DnaJ/GrpE	Takara Bio
pG-KJE8	Cm^R^, *P_araB_*::*dnaK-dnaJ-grpE* encoding DnaK/DnaJ/GrpE, *P_Pzt-1_*::*groES-groEL* encoding GroES/EL	Takara Bio

**Table 2:** Solubility of colicin M. Solubility percentages based on total and soluble yields of cell-free synthesized colicin M produced in various lysates and incubation temperatures and quantified by radioactive ^14^C-Leu scintillation counting (yield data shown in Figure 1A). BL21 Star (DE3) extracts enriched with chaperones by overexpression of GroES/EL (Gro), DnaK/DnaJ/GrpE (KJE), and both GroES/EL and DnaK/DnaJ/GrpE (Gro-KJE) were used. BL21 Star (DE3) extract without chaperone overexpression (Star) was used as a control. RT indicates room temperature (~25 °C). Average ± standard deviations are shown.

Extracts	Solubility (%)	
	30 °C	RT
Star	16 ± 4	16 ± 3
Gro	27 ± 2	30 ± 2
KJE	86 ± 3	80 ± 3
Gro-KJE	104 ± 2	102 ± 9

## Supplementary Materials

The following are available online at https://www.mdpi.com/2409-9279/2/2/28/s1, Table S1: Primers used in this study; Table S2: DNA sequences of all colicin genes; Figure S1: Overall CFPS capacity of chaperone-enriched cell extracts; Figure S2: Effect of additives in colicin E3 yield.

## References

[1] Blair J.M.A., Webber M.A., Baylay A.J., Ogbolu D.O., Piddock L.J.V. (2015). Molecular mechanisms of antibiotic resistance. Nat. Rev. Microbiol..

[2] Deak D., Outterson K., Powers J.H., Kesselheim A.S. (2016). Progress in the fight against multidrug-resistant bacteria? a review of U.S. Food and Drug Administration–approved antibiotics, 2010–2015. Ann. Intern. Med..

[3] Cotter P.D., Ross R.P., Hill C. (2013). Bacteriocins—A viable alternative to antibiotics?. Nat. Rev. Microbiol..

[4] Bahar A.A., Ren D. (2013). Antimicrobial peptides. Pharmaceuticals.

[5] Lee J.Y., Bang D. (2010). Challenges in the chemical synthesis of average sized proteins: Sequential vs. convergent ligation of multiple peptide fragments. Biopolymers.

[6] Salehi A.S.M., Smith M.T., Bennett A.M., Williams J.B., Pitt W.G., Bundy B.C. (2016). Cell-free protein synthesis of a cytotoxic cancer therapeutic: Onconase production and a just-add-water cell-free system. Biotechnol. J..

[7] Orth J.H.C., Schorch B., Boundy S., Ffrench-Constant R., Kubick S., Aktories K. (2011). Cell-free synthesis and characterization of a novel cytotoxic pierisin-like protein from the cabbage butterfly *Pieris rapae*. Toxicon.

[8] Martemyanov K.A., Shirokov V.A., Kurnasov O.V., Gudkov A.T., Spirin A.S. (2001). Cell-free production of biologically active polypeptides: Application to the synthesis of antibacterial peptide cecropin. Protein Expr. Purif..

[9] Jin X., Kightlinger W., Kwon Y.-C., Hong S.H. (2018). Rapid production and characterization of antimicrobial colicins using *Escherichia coli*-based cell-free protein synthesis. Synth. Biol..

[10] Jin X., Hong S.H. (2018). Cell-free protein synthesis for producing ‘difficult-to-express’ proteins. Biochem. Eng. J..

[11] Liu W.-Q., Zhang L., Chen M., Li J. (2019). Cell-free protein synthesis: Recent advances in bacterial extract sources and expanded applications. Biochem. Eng. J..

[12] Bundy B.C., Hunt J.P., Jewett M.C., Swartz J.R., Wood D.W., Frey D.D., Rao G. (2018). Cell-free biomanufacturing. Curr. Opin. Chem. Eng..

[13] Cascales E., Buchanan S.K., Duché D., Kleanthous C., Lloubès R., Postle K., Riley M., Slatin S., Cavard D. (2007). Colicin biology. Microbiol. Mol. Biol. Rev..

[14] El Ghachi M., Bouhss A., Barreteau H., Touzé T., Auger G., Blanot D., Mengin-Lecreulx D. (2006). Colicin M exerts its bacteriolytic effect via enzymatic degradation of undecaprenyl phosphate-linked peptidoglycan precursors. J. Biol. Chem..

[15] Zakharov S.D., Wang X.S., Cramer W.A. (2016). The colicin E1 TolC-binding conformer: Pillar or pore function of TolC in colicin import?. Biochemistry.

[16] Jakes K.S., Finkelstein A. (2010). The colicin Ia receptor, Cir, is also the translocator for colicin Ia. Mol. Microbiol..

[17] Sharma O., Yamashita E., Zhalnina M.V., Zakharov S.D., Datsenko K.A., Wanner B.L., Cramer W.A. (2007). Structure of the complex of the colicin E2 R-domain and its BtuB receptor. The outer membrane colicin translocon. J. Biol. Chem..

[18] Cramer W.A., Sharma O., Zakharov S.D. (2018). On mechanisms of colicin import: The outer membrane quandary. Biochem. J..

[19] González-Montalbán N., García-Fruitós E., Villaverde A. (2007). Recombinant protein solubility—Does more mean better?. Nat. Biotechnol..

[20] Kim Y.E., Hipp M.S., Bracher A., Hayer-Hartl M., Ulrich Hartl F. (2013). Molecular chaperone functions in protein folding and proteostasis. Annu. Rev. Biochem..

[21] Rosano G.L., Ceccarelli E.A. (2014). Recombinant protein expression in *Escherichia coli*: Advances and challenges. Front. Microbiol..

[22] Hartl F.U., Bracher A., Hayer-Hartl M. (2011). Molecular chaperones in protein folding and proteostasis. Nature.

[23] Niwa T., Kanamori T., Ueda T., Taguchi H. (2012). Global analysis of chaperone effects using a reconstituted cell-free translation system. Proc. Natl. Acad. Sci. USA.

[24] Stech M., Kubick S. (2015). Cell-free synthesis meets antibody production: A review. Antibodies.

[25] Fink A.L. (1999). Chaperone-mediated protein folding. Physiol. Rev..

[26] Oh I.-S., Lee J.-C., Lee M., Chung J., Kim D.-M. (2010). Cell-free production of functional antibody fragments. Bioprocess Biosyst. Eng..

[27] Kang S.-H., Kim D.-M., Kim H.-J., Jun S.-Y., Lee K.-Y., Kim H.-J. (2005). Cell-free production of aggregation-prone proteins in soluble and active forms. Biotechnol. Prog..

[28] Gopal G.J., Kumar A. (2013). Strategies for the production of recombinant protein in *Escherichia coli*. Protein J..

[29] Ahn J.-H., Chu H.-S., Kim T.-W., Oh I.-S., Choi C.-Y., Hahn G.-H., Park C.-G., Kim D.-M. (2005). Cell-free synthesis of recombinant proteins from PCR-amplified genes at a comparable productivity to that of plasmid-based reactions. Biochem. Biophys. Res. Commun..

[30] Jakes K.S. (2012). Translocation trumps receptor binding in colicin entry into *Escherichia coli*. Biochem. Soc. Trans..

[31] Kwon Y.-C., Jewett M.C. (2015). High-throughput preparation methods of crude extract for robust cell-free protein synthesis. Sci. Rep..

[32] Jakes K.S. (2017). The colicin E1 TolC box: Identification of a domain required for colicin E1 cytotoxicity and TolC binding. J. Bacteriol..

[33] Hong S.H., Ntai I., Haimovich A.D., Kelleher N.L., Isaacs F.J., Jewett M.C. (2014). Cell-free protein synthesis from a release factor 1 deficient *Escherichia coli* activates efficient and multiple site-specific non-standard amino acid incorporation. ACS Synth. Biol..

[34] Soelaiman S., Jakes K., Wu N., Li C., Shoham M. (2001). Crystal structure of colicin E3: Implications for cell entry and ribosome inactivation. Mol. Cell.

[35] Swartz J.R., Jewett M.C., Woodrow K.A. (2004). Cell-free protein synthesis with prokaryotic combined transcription-translation. Methods Mol. Biol..

[36] Sharma O., Cramer W.A. (2007). Minimum length requirement of the flexible N-terminal translocation subdomain of colicin E3. J. Bacteriol..

[37] Pédelacq J.-D., Cabantous S., Tran T., Terwilliger T.C., Waldo G.S. (2006). Engineering and characterization of a superfolder green fluorescent protein. Nat. Biotechnol..

[38] Acebrón S.P., Fernández-Sáiz V., Taneva S.G., Moro F., Muga A. (2008). DnaJ recruits DnaK to protein aggregates. J. Biol. Chem..

[39] Hayer-Hartl M., Bracher A., Hartl F.U. (2016). The GroEL-GroES chaperonin machine: A nano-cage for protein folding. Trends Biochem. Sci..

[40] Choi G.-H., Lee D.-H., Min W.-K., Cho Y.-J., Kweon D.-H., Son D.-H., Park K., Seo J.-H. (2004). Cloning, expression, and characterization of single-chain variable fragment antibody against mycotoxin deoxynivalenol in recombinant *Escherichia coli*. Protein Expr. Purif..

[41] Vera A., González-Montalbán N., Arís A., Villaverde A. (2007). The conformational quality of insoluble recombinant proteins is enhanced at low growth temperatures. Biotechnol. Bioeng..

[42] Jewett M.C., Fritz B.R., Timmerman L.E., Church G.M. (2013). *In vitro* integration of ribosomal RNA synthesis, ribosome assembly, and translation. Mol. Syst. Biol..

[43] Ng C.L., Lang K., Meenan N.A.G., Sharma A., Kelley A.C., Kleanthous C., Ramakrishnan V. (2010). Structural basis for 16S ribosomal RNA cleavage by the cytotoxic domain of colicin E3. Nat. Struct. Mol. Biol..

[44] Salehi A.S.M., Yang S.-O., Earl C.C., Shakalli Tang M.J., Porter Hunt J., Smith M.T., Wood D.W., Bundy B.C. (2018). Biosensing estrogenic endocrine disruptors in human blood and urine: A RAPID cell-free protein synthesis approach. Toxicol. Appl. Pharmacol..

[45] Zakharov S.D., Zhalnina M.V., Sharma O., Cramer W.A. (2006). The colicin E3 outer membrane translocon: Immunity protein release allows interaction of the cytotoxic domain with OmpF porin. Biochemistry.

[46] Smith M.T., Berkheimer S.D., Werner C.J., Bundy B.C. (2014). Lyophilized *Escherichia coli*-based cell-free systems for robust, high-density, long-term storage. Biotechniques.

[47] Hunt J.P., Yang S.O., Wilding K.M., Bundy B.C. (2017). The growing impact of lyophilized cell-free protein expression systems. Bioengineered.

[48] Nataro J.P., Kaper J.B. (1998). Diarrheagenic *Escherichia coli*. Clin. Microbiol. Rev..

[49] Bidell M.R., Palchak M., Mohr J., Lodise T.P. (2016). Fluoroquinolone and third-generation-cephalosporin resistance among hospitalized patients with urinary tract infections due to *Escherichia coli*: Do rates vary by hospital characteristics and geographic region?. Antimicrob. Agents Chemother..

[50] (1987). CIPRO^®^ (Ciprofloxacin Hydrochloride) [Product Information].

[51] Caschera F., Noireaux V. (2014). Synthesis of 2.3 mg/ml of protein with an all *Escherichia coli* cell-free transcription–translation system. Biochimie.

