# Supplementary material for: Optimizing Cell-Free Protein Synthesis for Increased Yield and Activity of Colicins
Source: Methods Protoc. 2019 Apr 11;2(2):28. doi: 10.3390/mps2020028 (PMC6632115; doi:10.3390/mps2020028)
Supplement: Supplementary file 1 [file mps-02-00028-s001.pdf]

Supplementary Information

Optimizing Cell-Free Protein Synthesis for Increased Yield and Activity of Colicins

Xing Jin,[1] Weston Kightlinger,[2] and Seok Hoon Hong[1,*]

[1]Department of Chemical and Biological Engineering,
Illinois Institute of Technology, Chicago, IL 60616, USA
[2]Department of Chemical and Biological Engineering,
Northwestern University, Evanston, IL 60208, USA

*Corresponding author: shong26@iit.edu
Tel: (+1) 312 567 8950

Supplementary Table S1. Primers used in this study. "*" indicates phosphorothioated bases.

Primer	Purpose	Sequence (5' → 3')
pJL1-F	Amplification of pJL1 backbone for Gibson Assembly	CAAAGCCCGAAAGGAAGCTGAGTTG
pJL1-R		CTTAAAGTTAAACAAAATTATTTCTAGAGGG
Gacol-F	Amplification of *cma* gene encoding colicin M	TTTGTTTAACTTTAAGAAGGAGATATACATATG
Gacol-R		TTCCTTTCGGGCTTTGTTAGCAGCCGGTCGACTTA
ColE1-F	Amplification of *cea* gene encoding colicin E1	ATTTTGTTTAACTTTAAGAAGGAGATATACATATGGAAACCGCGGTAGCGTAC
ColE1-R		CGGGCTTTGTTAGCAGCCGGTCGACTTATTTTTCGAACTGCGGATGGCTCCAAATCCCTAACACCTCATTTATAG
ColE3-F	Amplification of *ceaC* gene encoding colicin E3	ATTTTGTTTAACTTTAAGAAGGAGATATACATATGAGCGGTGGCGATGGACG
ColE3-R		CGGGCTTTGTTAGCAGCCGGTCGACTCATTTTTCGAACTGCGGATGGCTCCAAAGATATTTCTTGATATTTC
E3Imm-F	Amplification of *imm* gene encoding E3 immunity	ATTTTGTTTAACTTTAAGAAGGAGATATACATATGGGACTTAAATTGG
E3Imm-R		CGGGCTTTGTTAGCAGCCGGTCGACTCATTTTTCGAACTGCGGATGGCTCCACCAATCACCATC
T7Mega-F	Addition of T7 promoter	A*G*ATCTCGATCCCGCGAAATTAATACGACTCACTATAGGGAGACCACAACGGTTTCCCTCTAGAAATAATTTTGTTTAACTTTAAGAAGG
TAA-R	Addition of overlap region with T7 terminator	TATTGCTCAGCGGTGGCAGCAGCCAACTCAGCTTCCTTTCGGGCTTTGTTAGCAGCCGG
T7Mega-R	T7 terminator addition	T*A*ATCAGAATTGGCTTTCAGCAAAAAACCCCTCAAGACCCGTTTAGAGGCCCCAAGGGGTTATGCTAGTTATTGCTCAGCGGTGGCAGC
T7-Pro	Sequence checking for colicin amplification	TAATACGACTCACTATAGGG
T7-Ter		GCTAGTTATTGCCTCAGCGG

Supplementary Table S2. DNA sequences of all colicin genes. T7 promoter, terminator, colicin genes, and E3 immunity gene are highlighted as blue, red, turquoise, and green, respectively. Bolded lowercase characters indicate Strep-tag sequence.

Name	Size (bp)	Sequence 5’ → 3’
cma encoding M	1073	AGATCTCGATCCCGCGAAATTAATACGACTCACTATAGGGAGACCACAACGGTTTCCCTCTAGAAATA<br>ATTTTGTTTAACTTTAAGAAGGAGATATACATATGGAAACCTTAACTGTTCATGCACCATCACCATCA<br>ACTAACTTACCAAGTTATGGCAATGGTGCATTTTCTCTTTCAGCACCGCATGTTCCTGGTGCTGGACCT<br>CTTTTAGTCCAGGTTGTTTATAGTTTTTCCAGAGTCCAAACATGTGTCTTCAGGCTTTAACTCAACTT<br>GAGGATTACATCAAAAAACATGGGGCTAGCAACCCTCTCACATTGCAGATCATATCGACAAATATTG<br>GTTACTTCTGTAACGCCGACCGAAATCTGGTTCTTCACCCTGGAATAAGCGTTTATGACGCTTACCACT<br>TCTCAAAACCAGCGCCAAGTCAATATGACTATCGCTCAATGAATATGAAACAAATGAGCGGTAATGT<br>CACTACACCAATTGTGGCGCTTGCTCACTATTTATGGGGTAATGGCGCTGAAAGGAGCGTTAATATCG<br>CCAACATTGGTCTTAAAATTTCCCCTATGAAAATTAATCAGATAAAAGACATTATAAAATCTGGTGTA<br>GTAGGTACATTCCCTGTTTCTACAAAGTTCACACATGCCACTGGTGATTATAATGTTATTACCGGTGCA<br>TATCTTGGTAATATCACACTGAAAACAGAAGGTACTTTAACTATCTCTGCCAATGGCTCCTGGACTTA<br>CAATGGCGTTGTTCGTTCATATGATGATAAATACGATTTTAACGCCAGCACTCACCGTGGCGTCATCG<br>GAGAGTCGCTCACAAGGCTCGGGGCGATGTTTTCTGGTAAAGAGTACCAGATACTGCTTCCTGGTGAA<br>ATTCACATTAAAGAAAGTGGTAAGCGA**tggagccatccgcagttcgaaaaa**TAAGTCGACCGGCTGCTAACAAAG<br>CCCGAAAGGAAGCTGAGTTGGCTGCTGCCACCGCTGAGCAATAACTAGCATAACCCCTTGGGGCCTC<br>TAAACGGGTCTTGAGGGGTTTTTTGCTGAAAGCCAATTCTGATTA
cea encoding E1	1826	AGATCTCGATCCCGCGAAATTAATACGACTCACTATAGGGAGACCACAACGGTTTCCCTCTAGAAATA<br>ATTTTGTTTAACTTTAAGAAGGAGATATACATATGGAAACCGCGGTAGCGTACTATAAAGATGGTGTT<br>CCTTATGATGATAAGGGACAGGTAATTATTACTCTTTTGAATGGTACTCCTGACGGGAGTGGCTCTGG<br>CGGCGGAGGTGGAAAAGGAGGCAGTAAAAGTGAAAGTTCTGCAGCTATTCATGCAACTGCTAAATGG<br>TCTACTGCTCAATTAAAGAAAACACAGGCAGAGCAGGCTGCCCGGGCAAAAGCTGCAGCGGAAGCA<br>CAGGCGAAAGCAAAGGCAAACAGGGATGCGCTGACTCAGCGCCTGAAGGATATCGTGAATGAGGCT<br>CTTCGTCACAATGCCTCACGTACGCCTTCAGCAACAGAGCTTGCTCATGCTAATAATGCAGCTATGCA<br>GGCGGAAGACGAGCGTTTGCGCCTTGCGAAAGCAGAAGAAAAAGCCCGTAAAGAAGCGGAAGCAGC<br>AGAAAAGGCTTTTCAGGAAGCAGAACAACGACGTAAAGAGATTGAACGGGAGAAGGCTGAAACAGA<br>ACGCCAGTTGAAACTGGCTGAAGCTGAAGAGAAACGACTGGCTGCATTGAGTGAAGAAGCTAAAGCT<br>GTTGAGATCGCCCAAAAAAAACTTTCTGCTGCACAATCTGAAGTGGTGAAAATGGATGGAGAGATTA<br>AGACTCTCAATTCTCGTTTAAGCTCCAGTATCCATGCCCGTGATGCAGAAATGAAAACGCTCGCTGGA<br>AAACGAAATGAACTGGCTCAGGCATCCGCTAAATATAAAGAACTGGATGAGCTGGTCAAAAAACTAT<br>CACCAAGAGCCAATGATCCGCTTCAGAACCGTCCTTTTTTTGAAGCAACCAGACGACGGGTTGGGGCC<br>GGTAAGATTAGAGAAGAAAAACAAAAACAGGTAACAGCATCAGAAACACGTATTAACCGGATAAAT

		GCTGATATAACTCAGATCCAGAAGGCTATTTCTCAGGTCAGTAATAATCGTAATGCCGGTATCGCTCG<br>TGTTCATGAAGCTGAAGAAAATTTGAAAAAAGCACAGAATAATCTCCTTAATTCACAGATTAAGGAT<br>GCTGTTGATGCAACAGTTAGCTTTTATCAAACGCTGACTGAAAAATATGGTGAAAAATATTCGAAAAT<br>GGCACAGGAACTTGCTGATAAGTCTAAAGGTAAGAAAATCGGCAATGTGAATGAAGCTCTCGCTGCT<br>TTTGAAAAATACAAGGATGTTTTAAATAAGAAATTCAGCAAAGCCGATCGTGATGCTATTTTTAATGC<br>GTTGGCATCGGTGAAGTATGATGACTGGGCTAAACATTTAGATCAGTTTGCCAAGTACTTGAAGATTA<br>CGGGGCATGTTTCTTTTGGATATGATGTGGTATCTGATATCCTAAAAATTAAGGATACAGGTGACTGG<br>AAGCCACTATTTCTTACATTAGAGAAGAAAGCTGCAGATGCAGGGGTGAGTTATGTTGTTGCTTTACT<br>TTTTAGCTTGCTTGCTGGAACTACATTAGGTATTTGGGGTATTGCTATTGTTACAGGAATTCTATGCTC<br>CTATATTGATAAGAATAAACTTAATACTATAAATGAGGTGTTAGGGATT**tggagccatccgcagttcgaaaaa**TAA<br>GTCGACCGGCTGCTAACAAAGCCCGAAAGGAAGCTGAGTTGGCTGCTGCCACCGCTGAGCAATAACT<br>AGCATAACCCCTTGGGGCCTCTAAACGGGTCTTGAGGGGTTTTTTGCTGAAAGCCAATTCTGATTA
ceaC encoding E3	1889	AGATCTCGATCCCGCGAAATTAATACGACTCACTATAGGGGAGACCACAACGGTTTCCCTCTAGAAATA<br>ATTTTGTTTAACTTTAAGAAGGAGATATACATATGAGCGGTGGCGATGGACGCGGCCATAACACGGG<br>CGCGCATAGCACAAGTGGTAACATTAATGGTGGCCCGACCGGGCTTGGTGTAGGTGGTGGTGCTTCTG<br>ATGGCTCCGGATGGAGTTCGGAAAATAACCCGTGGGGTGGTGGTTCCGGTAGCGGCATTCACTGGGG<br>TGGTGGTTCCGGTCATGGTAATGGCGGGGGGAATGGTAATTCCGGTGGTGGTTCGGGAACAGGCGGT<br>AATCTGTCAGCAGTAGCTGCGCCAGTGGCATTTGGTTTTCCGGCACTTTCCACTCCAGGAGCTGGCGG<br>TCTGGCGGTCAGTATTTCAGCGGGAGCATTATCGGCAGCTATTGCTGATATTATGGCTGCCCTGAAAG<br>GACCGTTTAAATTTGGTCTTTGGGGGGTGGCTTTATATGGTGTATTGCCATCACAAATAGCGAAAGAT<br>GACCCCAATATGATGTCAAAGATTGTGACGTCATTACCCGCAGATGATATTACTGAATCACCTGTCAG<br>TTCATTACCTCTCGATAAGGCAACAGTAAACGTAAATGTTCGTGTTGTTGATGATGTAAAAGACGAGC<br>GACAGAATATTTCGGTTGTTTCAGGTGTTCCGATGAGTGTTCCGGTGGTTGATGCAAAACCTACCGAA<br>CGTCCGGGTGTTTTTACGGCATCAATTCCAGGTGCACCTGTTCTGAATATTTCAGTTAATAACAGTACG<br>CCAGCAGTACAGACATTAAGCCCAGGTGTTACAAATAATACTGATAAGGATGTTCGCCCGGCAGGAT<br>TTACTCAGGGTGGTAATACCAGGGATGCAGTTATTCGATTCCCGAAGGACAGCGGTCATAATGCCGTA<br>TATGTTTCAGTGAGTGATGTTCTTAGCCCTGACCAGGTAAAACAACGTCAAGATGAAGAAAATCGCC<br>GTCAGCAGGAATGGGATGCTACGCATCCGGTTGAAGCGGCTGAGCGAAATTATGAACGCGCGCGTGC<br>AGAGCTGAATCAGGCAAATGAAGATGTTGCCAGAAATCAGGAGCGACAGGCTAAAGCTGTTCAGGTT<br>TATAATTCGCGTAAAAGCGAACTTGATGCAGCGAATAAAACTCTTGCTGATGCAATAGCTGAAATAA<br>AACAATTTAATCGATTTGCCCATGACCCAATGGCTGGCGGTCACAGAATGTGGCAAATGGCCGGGCTT<br>AAAGCCCAGCGGGCGCAGACGGATGTAAATAATAAGCAGGCTGCATTTGATGCTGCTGCAAAAGAGA<br>AGTCAGATGCTGATGCTGCATTGAGTTCTGCTATGGAAAGCAGGAAGAAGAAAGAAGATAAGAAAA<br>GGAGTGCTGAAAATAATTTAAACGATGAAAAGAATAAGCCCAGAAAAGGTTTTAAAGATTACGGGCA<br>TGATTATCATCCAGCTCCGAAAACTGAGAATATTAAAGGGCTTGGTGATCTTAAGCCTGGGATACCAA<br>AAACACCAAAGCAGAATGGTGGTGGAAAACGCAAGCGCTGGACTGGAGATAAAGGGCGTAAGATTT

		ATGAGTGGGATTCTCAGCATGGTGAGCTTGAGGGGTATCGTGCCAGTGATGGTCAGCATCTTGGCTCA<br>TTTGACCCTAAAACAGGCAATCAGTTGAAAGGTCCAGATCCGAAACGAAATATCAAGAAATATCTTT<br>GAGTCGACCGGCTGCTAACAAAGCCCGAAAGGAAGCTGAGTTGGCTGCTGCCACCGCTGAGCAATAA<br>CTAGCATAACCCCTTGGGGCCTCTAAACGGGTCTTGAGGGGTTTTTTGCTGAAAGCCAATTCTGATTA
E3+Imm operon	2156	AGATCTCGATCCCGCGAAATTAATACGACTCACTATAGGGAGACCACAACGGTTTCCCTCTAGAAATA<br>ATTTTGTTTAACTTTAAGAAGGAGATATACATATGAGCGGTGGCGATGGACGCGGCCATAACACGGG<br>CGCGCATAGCACAAGTGGTAACATTAATGGTGGCCCGACCGGGCTTGGTGTAGGTGGTGGTGCTTCTG<br>ATGGCTCCGGATGGAGTTCGGAAAATAACCCGTGGGGTGGTGGTTCCGGTAGCGGCATTCACTGGGG<br>TGGTGGTTCCGGTCATGGTAATGGCGGGGGGAATGGTAATTCCGGTGGTGGTTCGGGAACAGGCGGT<br>AATCTGTCAGCAGTAGCTGCGCCAGTGGCATTTGGTTTTCCGGCACTTTCCACTCCAGGAGCTGGCGG<br>TCTGGCGGTCAGTATTTCAGCGGGAGCATTATCGGCAGCTATTGCTGATATTATGGCTGCCCTGAAAG<br>GACCGTTTAAATTTGGTCTTTGGGGGGTGGCTTTATATGGTGTATTGCCATCACAAATAGCGAAAGAT<br>GACCCCAATATGATGTCAAAGATTGTGACGTCATTACCCGCAGATGATATTACTGAATCACCTGTCAG<br>TTCATTACCTCTCGATAAGGCAACAGTAAACGTAAATGTTCGTGTTGTTGATGATGTAAAAGACGAGC<br>GACAGAATATTTCGGTTGTTTCAGGTGTTCCGATGAGTGTTCCGGTGGTTGATGCAAAACCTACCGAA<br>CGTCCGGGTGTTTTTACGGCATCAATTCCAGGTGCACCTGTTCTGAATATTTCAGTTAATAACAGTACG<br>CCAGCAGTACAGACATTAAGCCCAGGTGTTACAAATAATACTGATAAGGATGTTCGCCCGGCAGGAT<br>TTACTCAGGGTGGTAATACCAGGGATGCAGTTATTCGATTCCCGAAGGACAGCGGTCATAATGCCGTA<br>TATGTTTCAGTGAGTGATGTTCTTAGCCCTGACCAGGTAAAACAACGTCAAGATGAAGAAAATCGCC<br>GTCAGCAGGAATGGGATGCTACGCATCCGGTTGAAGCGGCTGAGCGAAATTATGAACGCGCGCGTGC<br>AGAGCTGAATCAGGCAAATGAAGATGTTGCCAGAAATCAGGAGCGACAGGCTAAAGCTGTTCAGGTT<br>TATAATTCGCGTAAAAGCGAACTTGATGCAGCGAATAAAACTCTTGCTGATGCAATAGCTGAAATAA<br>AACAATTTAATCGATTTGCCCATGACCCAATGGCTGGCGGTCACAGAATGTGGCAAATGGCCGGGCTT<br>AAAGCCCAGCGGGCGCAGACGGATGTAAATAATAAGCAGGCTGCATTTGATGCTGCTGCAAAAGAGA<br>AGTCAGATGCTGATGCTGCATTGAGTTCTGCTATGGAAAGCAGGAAGAAGAAAGAAGATAAGAAAA<br>GGAGTGCTGAAAATAATTTAAACGATGAAAAGAATAAGCCCAGAAAAGGTTTTAAAGATTACGGGCA<br>TGATTATCATCCAGCTCCGAAAACTGAGAATATTAAAGGGCTTGGTGATCTTAAGCCTGGGATACCAA<br>AAACACCAAAGCAGAATGGTGGTGGAAAACGCAAGCGCTGGACTGGAGATAAAGGGCGTAAGATTT<br>ATGAGTGGGATTCTCAGCATGGTGAGCTTGAGGGGTATCGTGCCAGTGATGGTCAGCATCTTGGCTCA<br>TTTGACCCTAAAACAGGCAATCAGTTGAAAGGTCCAGATCCGAAACGAAATATCAAGAAATATCTTT<br>GAGAGGAAGTTATGGGACTTAAATTGGATTTAACTTGGTTTGATAAAAGTACAGAAGATTTTAAGGG<br>TGAGGAGTATTCAAAAGATTTTGGAGATGACGGTTCAGTTATGGAAAGTCTAGGTGTGCCTTTTAAGG<br>ATAATGTTAATAACGGTTGCTTTGATGTTATAGCTGAATGGGTACCTTTGCTACAACCATACTTTAATC<br>ATCAAATTGATATTTCCGATAATGAGTATTTTGTTTCGTTTGATTATCGTGATGGTGATTGGTGAGTCG<br>ACCGGCTGCTAACAAAGCCCGAAAGGAAGCTGAGTTGGCTGCTGCCACCGCTGAGCAATAACTAGCA<br>TAACCCCTTGGGGCCTCTAAACGGGTCTTGAGGGGTTTTTTGCTGAAAGCCAATTCTGATTA

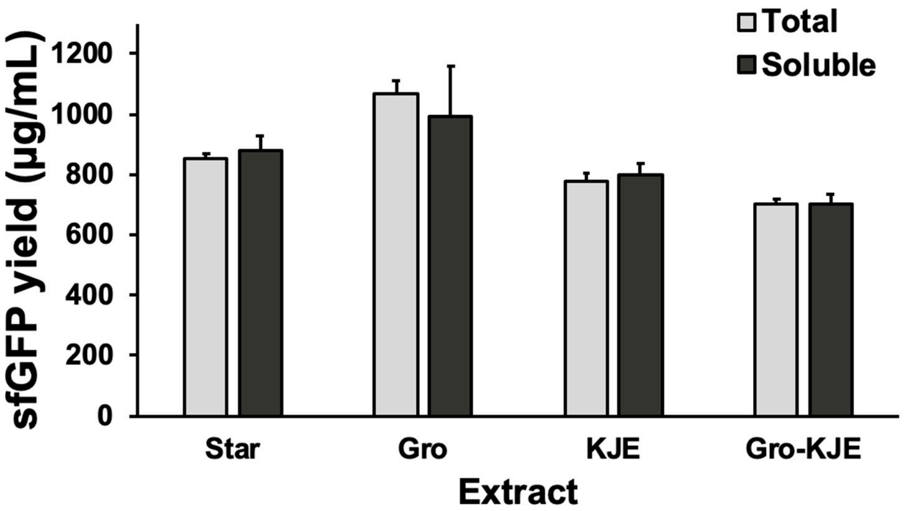

Supplementary Figure S1. Overall CFPS capacity of chaperone-enriched extracts. Total and soluble protein yields of sfGFP produced in chaperone-enriched extracts determined by ^{14}C-Leu scintillation counting at 30°C for 20 h.

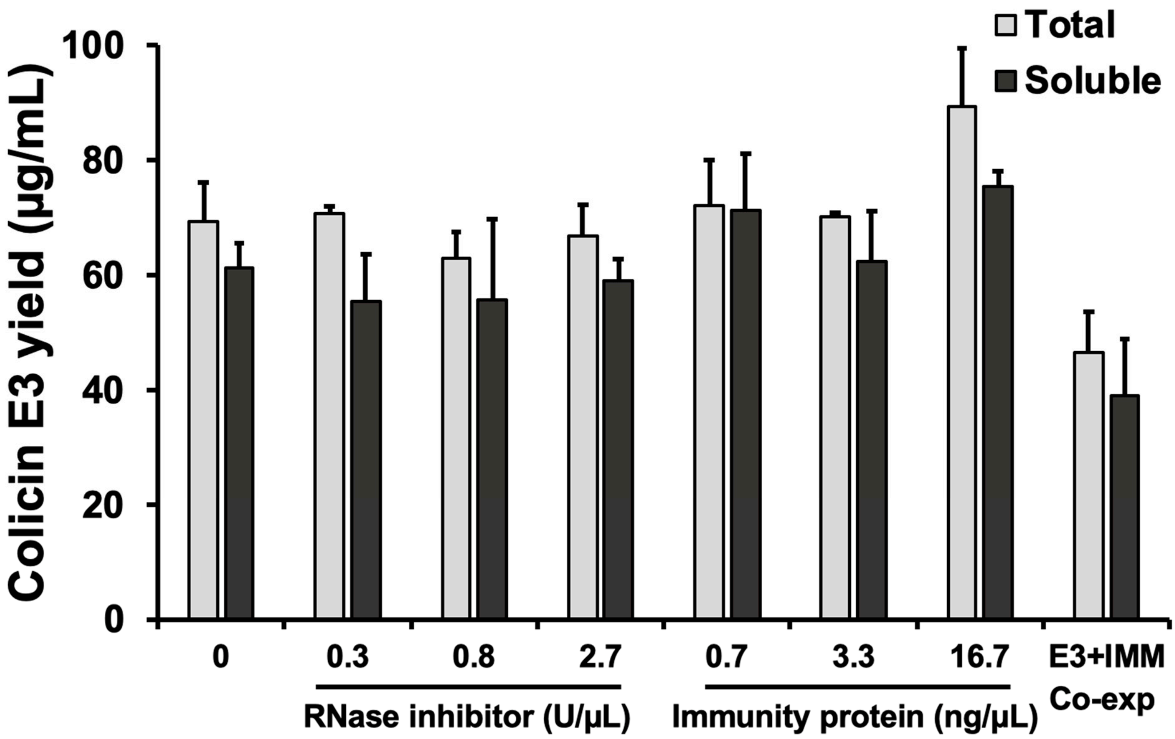

Supplementary Figure S2. Effect of additives in colicin E3 yield. Yield of cell-free synthesized colicin E3 with different concentrations of RNase inhibitor and E3 immunity protein and with co-expression (co-exp) of immunity protein. Error bars indicate standard deviation from three independent CFPS reactions.
